# Circular RNA circ_0000228 promotes the malignancy of cervical cancer via microRNA-195-5p/ lysyl oxidase-like protein 2 axis

**DOI:** 10.1080/21655979.2021.1954846

**Published:** 2021-07-24

**Authors:** Shimei Liu, Bingqing Li, Ying Li, Huaihua Song

**Affiliations:** Department of Obstetrics and Gynecology, The Third People’s Hospital of Linyi, Linyi, Shandong, China

**Keywords:** Cervical cancer, circ_0000228, miR-195-5p, LOXL2

## Abstract

Circular RNAs (circRNAs) are a class of novel non-coding RNAs that are vital in modulating gene expression and biological processes. Nevertheless, in cervical cancer (CC), the role of circRNA is much less investigated. In this work, circ_0000228 expression in CC is measured and circ_0000228’s function and related mechanism are investigated. Quantitative real-time quantitative polymerase chain reaction (qRT-PCR) was utilized to examine the expression levels of circ_0000228, microRNA-195-5p (miR-195-5p) and lysyl oxidase-like protein 2 (LOXL2). Western blotting was employed to examine LOXL2 protein expression in CC cell lines. CC cell lines with circ_0000228 knockdown were constructed, and the CCK-8 experiment and Transwell experiment were executed to investigate the effect of circ_0000228 on the malignant characteristics of CC cells. Furthermore, a dual-luciferase reporter gene experiment was applied to validate the targeting relationship between circ_0000228 and miR-195-5p, miR-195-5p and LOXL2. In this study, we demonstrated that circ_0000228 showed a remarkable up-modulation in CC tissues and cell lines. Circ_0000228 knockdown repressed the growth and metastatic potential of CC cells. Mechanistically, circ_0000228 facilitated CC progression through sponging miR-195-5p and up-modulating LOXL2 expression. We conclude that circ_0000228 is an oncogenic circRNA, which participates in promoting CC progression via regulating the miR-195-5p/LOXL2 axis.

## GRAPHICAL ABSTRACT

**Figure:**
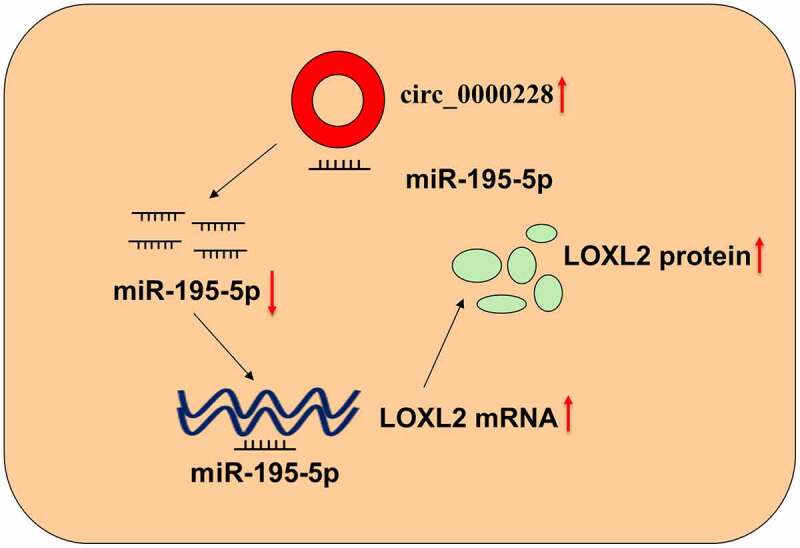

## Introduction

Cervical cancer (CC) is a common malignancy among women worldwide [1]. Human papillomavirus infection is the major risk factor of CC [2]. Although significant progress has been made in surgery, chemotherapy or radiotherapy, the 5-year survival rate for patients in developing countries is still below 30% [3]. CC development is modulated by multiple molecules, and understanding the molecular mechanisms is essential for the discovery of innovative biomarkers and therapy targets for this disease.

Circular RNAs (circRNAs) are non-coding RNAs with covalently linked, closed-loop structures [4]. In recent years, circRNAs have attracted widespread attention in cancer research. Increasing research implies that circRNAs may be promising biomarkers and therapeutic targets for tumors [5]. Diverse circRNAs are aberrantly expressed in CC and are implicated in tumorigenesis and metastasis, such as circ_0018289, circEIF4G2, and circCLK3 [4]. Nevertheless, the function of the majority of circRNAs in CC remains blurred. In this work, we demonstrate that circ_0000228 (circ_002044), which is produced by the transcript of zinc finger E-box binding homeobox 1 (ZEB1), is remarkably up-modulated in CC by analyzing microarray data from Gene Expression Omnibus (GEO) database. Nonetheless, its accurate function and related mechanism in CC are undefined.

MicroRNAs (miRNAs) are implicated in tumorigenesis by binding to the 3’ UTR of target mRNA and repressing their translation. Diverse miRNAs can participate in CC development as tumor-suppressive miRNAs or oncomiRs [6–9]. For instance, miR-145-5p targets fascin to impede cancer cell migration and invasion in CC [6]. Lysyl oxidase-like protein 2 (LOXL2) belongs to the lysine oxidase gene family and is crucial in catalyzing the formation of elastin and collagen cross-links in the extracellular matrix (ECM) [10]. LOXL2 overexpression is reported to accelerate CC progression [11]. Nevertheless, the mechanism of LOXL2 dysregulation in CC is unclear.

This work is aimed to explore the expression characteristics and biological functions of circ_0000228 in CC. We hypothesized that circ_0000228 is an oncogenic circRNA in CC. Herein, we report that circ_0000228 is markedly up-regulated in CC tissues and cell lines, and it can adsorb miR-195-5p to up-modulate LOXL2 expression to facilitate CC progression.

## Materials and methods

### Subjects and tissue specimens

Forty CC tissues and 40 paracancerous tissues were available from the patients of the Third People’s Hospital of Linyi from May 2017 to May 2019. All patients were diagnosed by histopathological biopsy, and all the samples were confirmed by the pathologists after surgery. None of the subjects had undergone radiotherapy, chemotherapy, or other treatment before surgery. The 40 CC patients were staged according to the AJCC criteria, and 21 and 19 cases were classified into stage I and II, respectively. The specimens were preserved in liquid nitrogen immediately after removal. The work was endorsed by the Ethics Committee of the Third People’s Hospital of Linyi and the procedures followed the Declaration of Helsinki.

### Cell culture

Four human CC cell lines (HeLa, HCC94, SW756, and C33A) and human normal cervical epithelial cells (HUCEC) were procured from the American Type Culture Collection (ATCC, Bethesda, MD, USA) and the Chinese Academy of Sciences Committee Type Culture Collection Cell Bank (Shanghai, China). All cells were routinely cultured in RPMI-1640 medium (Sangon, Shanghai, China) containing 10% fetal bovine serum (FBS) (Gibco, NY, USA), penicillin (100 U/ml, Gibco, NY, USA) and streptomycin (100 μg/ml, Gibco, NY, USA) at 37°C in 5% CO_2_ [12].

### Cell transfection

Circ_0000228 (si-circ_0000228#1 and si-circ_0000228#2) and negative control siRNA (si-NC), miR-195-5p mimics (miR-195-5p) and miR-195-5p inhibitors (miR-195-5p in) and their corresponding controls was synthesized by RiboBio (Guangzhou, China). When the cells reached 80% confluence, the plasmids or oligonucleotides were transfected into the cells using Lipofectamine^TM^ 2000 (Invitrogen, Carlsbad, CA, USA) [13].

### Quantitative real-time quantitative polymerase chain reaction (qRT-PCR)

Total RNA was isolated from tissues and cell lines using TRIzol reagent (ThermoFisher Scientific, Waltham, MA, USA), and total RNA was reversely transcribed to cDNA using the PrimeScript™ RT reagent kit (Takara, Dalian, China) [13]. With the cDNA as the template, qRT-PCR was performed with SYBR® Premix Ex Taq^TM^ II Kit (TaKaRa, Dalian, China) on StepOnePlus Real-Time PCR System (ThermoFisher Scientific, Waltham, MA, USA). After the amplification reaction, the relative expression of the genes were calculated using 2^−ΔΔCt^ method, with GAPDH and U6 as internal references. The primer sequences were as follows:

circ_0000228, 5’-TGAGCTTGTGAGTGAGTGGT-3’ (forward) and 5’-GCAAGGAGAATGGCGAGATG-3’; (reverse); miR-195-5p, 5’- ACACTCCAGCTGGGTAGCAGCACAGAAAT-3’ (forward) and 5’- TGGTGTCGTGGAGTCG-3’; (reverse)); LOXL2, 5’-GGCACCGTGTGCGATGACGA-3’ (forward), and 5’- GCTGCAAGGGTCGCCTCGTT-3’; (reverse); ZEB1, 5’-AGCGAGGTAAAGTTGCGTCT-3’ (forward), and 5’-AGGTTTTCTGGGCCCCG-3’ (reverse); U6, 5’-GACTATCATATGCTTACCGT-3’ (forward) and 5’-GGGCAGGAAGAGGGCCTAT-3’ (reverse); GAPDH, 5’-CTTTGGTATCGTGGAAGGACTC-3’ (forward) and 5’;-GTAGAGGCAGGGATGATGTTCT-3’ (reverse).

### Subcellular fractionation

Briefly, RNA was isolated from the nucleus and cytoplasm of HCC94 and C33A cells using the PARIS™ Kit (ThermoFisher Scientific, Waltham, MA, USA), respectively [14]. Circ_0000228 and ZEB1 mRNA expression in nuclear and cytoplasmic RNA was detected by qRT-PCR. U6 was a nuclear marker and GAPDH was a cytoplasmic marker.

### Cell counting kit-8 (CCK-8) experiment

The CC cells were inoculated into 96-well plates (2 × 10^3^ cells/well), and routinely cultured. Ten μL of CCK-8 solution (Dojindo Laboratories, Kumamoto, Japan) was supplemented to each well on days 1, 2, 3 and 4, respectively. After the CCK-8 solution was added, the cells were incubated at 37°C for 2 h [15]. Next, the absorbance of the cells at 450 nm wavelength was measured using a microplate reader (Bio-Rad, Hercules, CA, USA).

### Transwell experiment

Transwell chambers (Corning, MA, USA) with or without Matrigel were adopted for cell migration and invasion experiments [13]. For cell migration experiments: 1 × 10^5^ cells were resuspended in 150 µL of serum-free medium and transferred in the upper compartment of the Transwell chambers. After the cells were cultured at 37°C for 24 h, the cells on the upper compartment were wiped away, and the cells on the lower surface of the filter were fixed with 95% ethanol and stained with 0.1% crystal violet for 25 min. Next, a microscope (Olympus, Japan) was applied for counting the cells. The cell invasion experiment was performed with the Transwell chambers whose filters were pre-coated with a layer of Matrigel, the rest of the procedures were the same as the cell migration experiment.

### Dual-luciferase reporter gene experiment

Wild-type (WT) and mutant (MUT) sequences of circ_0000228 or LOXL2 3’ UTR with potential complementary binding sequences to miR-195-5p were synthesized by Promega (Madison, WI, USA) and cloned into psiCHECK^TM^-2-luciferase vector (Promega, Madison, WI, USA). The luciferase reporter plasmid was then co-transfected with miR-195-5p mimics or miR-NC into 293 T cells, and 48 h later, the dual-luciferase reporter assay system (Promega, Madison, WI, USA) was used for measuring the relative luciferase activity of the cells in different groups [16].

### Western blot

The total protein was isolated from the cells using RIPA lysis buffer (Beyotime, Shanghai, China) and protein concentration was quantified using a BCA kit (Beyotime, Shanghai, China) [17]. SDS-PAGE was used to dissolve the protein samples (30 μg/lane), and then the protein was transferred to polyvinylidene fluoride (PVDF) membranes (Millipore, Billerica, MA, USA). Then, the PVDF membranes were blocked with 5% skim milk at room temperature for 1 h, and incubated with primary antibodies including anti-LOXL2 (1:1000, ab96233, Abcam Inc., Cambridge, UK), anti-GAPDH (1:1000, ab9485, Abcam Inc., Cambridge, UK) at 4°C overnight. Next, the membranes were incubated with horseradish peroxidase-conjugated secondary antibody (1:2000, ab150077, Abcam Inc., Cambridge, UK) for 1 h at room temperature, and finally, the protein bands were detected using an ECL kit (ThermoFisher Scientific, Waltham, MA, USA).

### Bioinformatics analysis

The GEO dataset GSE113696 contained the circRNA expression profile data of one normal cervical epithelial cell line and 5 CC cell lines. GSE86100 contained miRNA expression profile data of six cases of normal mucosa tissues and six cases of CC tissues. The two datasets were downloaded from GEO database (https://www.ncbi.nlm.nih.gov/gds) for identifying the differentially expressed circRNAs or miRNAs in CC. The target miRNAs of circ_0000228 were predicted by StarBase database (http://starbase.sysu.edu.cn/). The Venn Diagram web tool (http://bioinformatics.psb.ugent.be/webtools/Venn) was used to screen out the miRNAs, which contain potential complementary binding sites with circ_0000228.

### Statistical analysis

All experiments were performed in triplicate for 3 times, and the data were expressed as mean ± standard deviation. Statistical analysis was performed using SPSS 17.0 software (SPSS Inc., Chicago, IL, USA) and the figures were plotted using GraphPad Prism V5.0 (GraphPad Software, Inc., La Jolla, CA, USA). Student’s *t*-test was employed for two-group analysis, and one-way ANOVA was applied for three or more groups. Pearson’s correlation was adopted for correlation analysis. *p < 0.05, **p < 0.01 and ***p < 0.001 signified statistical significance.

## Results

Our study is aimed to explore the expression, function and potential mechanism of circ_0000228 in CC. We hypothesized that circ_0000228 could promote the malignancy of CC cells. In this work, with *in vitro* experiments, we identified a novel circRNA, circ_0000228, which was remarkably up-modulated in CC tissues and cell lines, adsorbed miR-195-5p to up-modulate LOXL2 expression to facilitate CC progression.

### Circ_0000228 was remarkably up-regulated in CC tissues and cell lines

**Figure 1.**
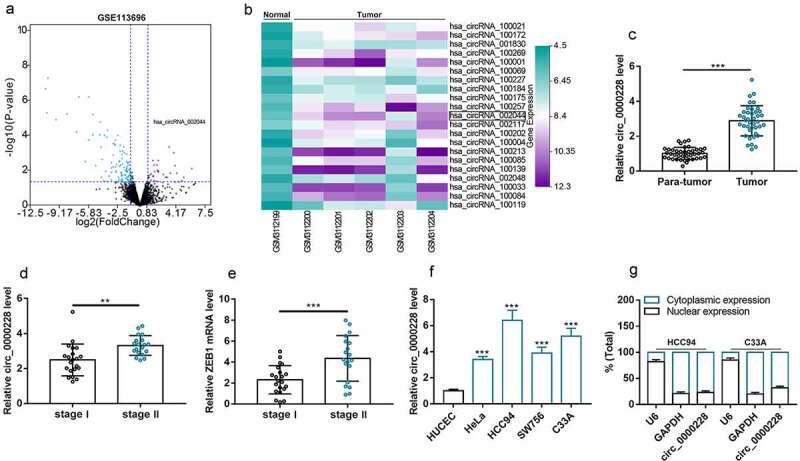
Circ_0000228 was remarkably up-modulated in CC tissues and cell lines

The circRNA microarray GSE113696 was downloaded from the GEO database, and the cutoff criteria were as follows: | log2 (fold change) |>1 and *P* < 0.05, which was used to screen out the differentially expressed circRNAs in CC cell lines (Figure 1(a)). The expression profiles of the 21 circRNAs that were markedly up-modulated in the CC cell lines were displayed in Figure 1(b). Then, circ_0000228, which was significantly up-regulated in the CC lines (circ_002044; log2 (fold change) = 3.44 and *p* < 0.001) was selected for further analysis. Circ_0000228 expression in 40 pairs of CC tissues and paracancerous tissues was measured using qRT-PCR, and the data showed that circ_0000228 expression was remarkably augmented in CC tissues relative to paracancerous tissues (Figure 1(c)). Then, we compared the expression of circ_0000228 and ZEB1 mRNA in CC tissues of 21 patients with stage I CC and 19 patients with stage II CC. The results showed that circ_0000228 and ZEB1 expression levels were significantly increased in stage II CC patients compared with stage 1 CC patients (Figure 1(d,e)). Additionally, the data of qRT-PCR also showed that circ_0000228 expression was remarkably elevated in CC cell lines (HeLa, HCC94, SW756, C33A) relative to HUCEC lines (Figure 1(e)), which is consistent with the results of microarray. Given that circ_0000228 was expressed at the highest levels in HCC94 and C33A cell lines, these two cell lines were selected for follow-up assays. The cytoplasm and nucleus of CC cells were then isolated to determine the subcellular distribution of circ_0000228. The data of qRT-PCR indicated that circ_0000228 was mainly distributed in the cytoplasm of HCC94 and C33A cells, suggesting that circ_0000228 could probably function as a competitive endogenous RNA (ceRNA) (Figure 1(f)).

(a) A volcano plot was employed to analyze the differentially expressed circRNAs in the GEO dataset (GSE113696) that were remarkably up- and down-modulated in CC cell lines, compared with normal cervical epithelial cells; (b) A heat maps was utilized to present the significantly highly expressed circRNAs in CC; (c) Circ_0000228 expression in 40 CC tissues/paired adjacent cervical tissues was examined by qRT-PCR; (d) Circ_0000228 expression in 21 patients with stage I CC and 19 patients with stage II CC was analyzed by qRT-PCR, respectively; (e) ZEB1 mRNA expression in 21 patients with stage I CC and 19 patients with stage II CC was analyzed by qRT-PCR, respectively; (f) Circ_0000228 expression in HUCEC and CC cell lines (HeLa, HCC94, SW756, C33A) was detected by qRT-PCR; (g) Circ_0000228 expression in the nucleus and cytoplasm of HCC94 and C33A cells was detected by qRT-PCR. All of the experiments were performed in triplicate. ***P < 0.001.

### Knockdown of circ_0000228 remarkably repressed CC cell multiplication, migration, and invasion

**Figure 2.**
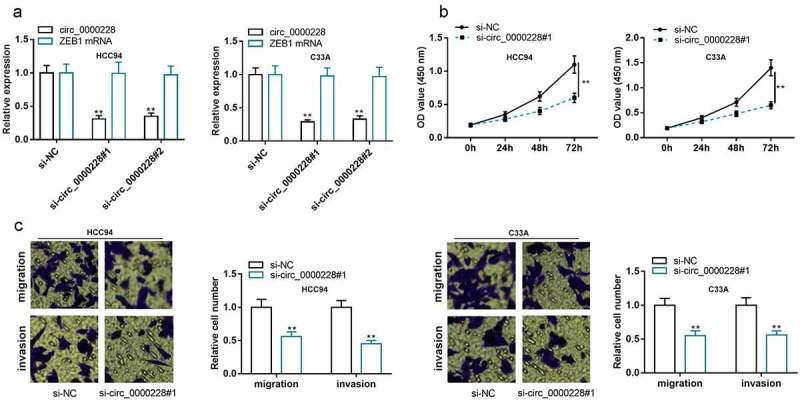
Knockdown of circ_0000228 remarkably impeded CC cell multiplication, migration, and invasion

To probe the role of circ_0000228 in regulating the biological behaviors of CC cells, two circ_0000228 siRNAs (si-circ_0000228#1 and si-circ_0000228#2) were transfected into HCC94 and C33A cell lines, and circ_0000228 knockdown models were successfully constructed: after the CC cells were transfected with the siRNAs, circ_0000228 expression was markedly suppressed, whereas the linear ZEB1 mRNA expression was unchanged (Figure 2(a)). Considering si-circ_0000228#1 showed higher knockdown efficiency, it was used for the subsequent experiments. CCK-8 experiment was employed to examine the proliferation of CC cells, and it was demonstrated that the cell growth in the circ_0000228 knockdown group was remarkably lower than that in the control group in both cell lines (Figure 2(b)). Transwell experiments were executed to measure cell migration and invasion, and the data suggested that CC cells’ migration and invasion were remarkably suppressed in the circ_0000228 knockdown group relative to the control group in both cell lines (Figure 2(c)). The above data implied that circ_0000228 facilitated the malignant phenotypes of CC cells.

(a) qRT-PCR was executed to detect circ_0000228 and ZEB1 mRNA expression in CC cells transfected with circ_0000228 siRNA; (b) CCK-8 experiment was conducted to detect the multiplication of CC cells after transfection with circ_0000228 siRNA; (c) Transwell experiment was employed to detect migration and invasion of CC cells after transfection with circ_0000228 siRNA. All of the experiments were performed in triplicate. **P < 0.01 and ***P < 0.001.

### Circ_0000228 positively regulated LOXL2 expression through repressing miR-195-5p

**Figure 3.**
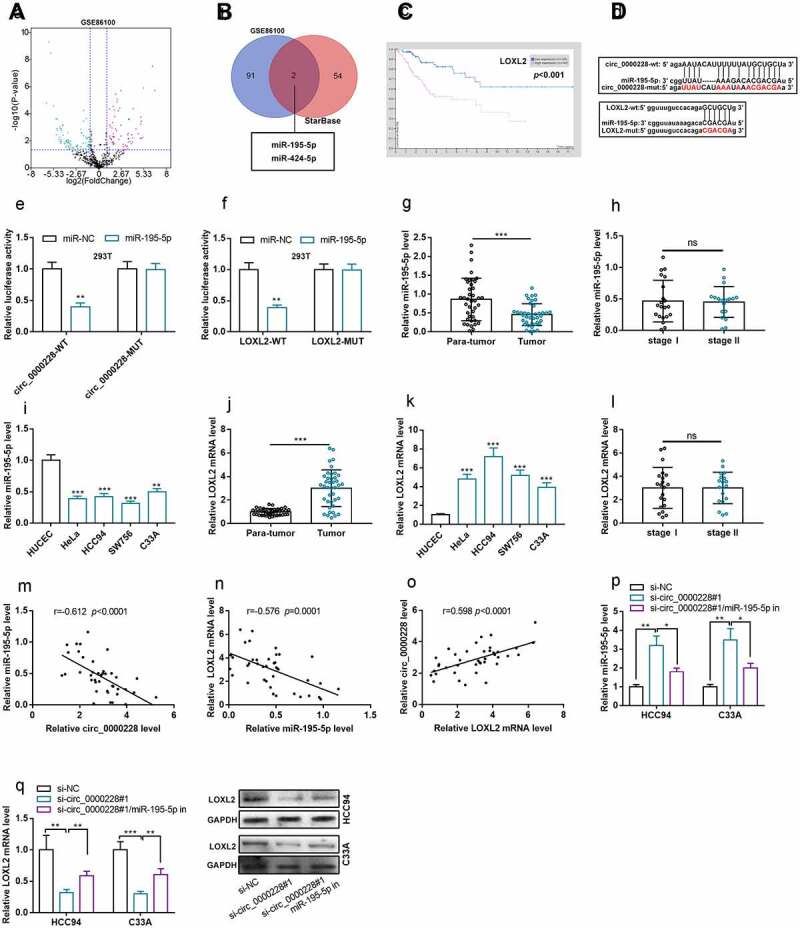
Circ_0000228 modulated LOXL2 expression through miR-195-5p

Aberrantly expressed miRNAs in CC tissues were screened out by analyzing GEO dataset GSE86100 (Figure 3(a)). The StarBase database was searching for predicting the downstream miRNAs of circ_0000228. Venn diagram then identified two miRNAs that were remarkably down-modulated at GSE86100 and had complementary potential binding sites with circ_0000228, namely miR-195-5p [log2 (fold change) = −2.18 and *p* < 0.001] and miR-424-5p [log2 (fold change) = −1.23 and *p* < 0.001] (Figure 3(b)). Additionally, high expression of LOXL2 was associated with to shorter survival time of CC patients in Human Protein ATLAS database (Figure 3(c)). Interestingly, LOXL2 was predicted as a potential target gene of miR-195-5p by StarBase database (Figure 3(d)). To validate the binding relationships among circ_0000228, miR-195-5p and LOXL2, wild type and mutant-type luciferase reporter vectors carrying circ_0000228 sequence or LOXL2 3ʹ-untranslated region (3ʹUTR) sequence were designed and constructed (Figure 3(d)), and dual-luciferase reporter gene experiments were performed. The data showed that miR-195-5p remarkably repressed the luciferase activity of circ_0000228-WT or LOXL2-WT in 293 T cells, but exerted no significant effect on that of circ_0000228-MUT or LOXL2-MUT (Figure 3(e,f)). Besides, qRT-PCR was employed to determine miR-195-5p expression in CC tissues and cell lines. As shown, miR-195-5p was remarkably down-modulated in CC tissues and cell lines; however, miR-195-5p expression was not associated with the TNM stage of the tumors (Figure 3(g–i)). qRT-PCR also showed that, LOXL2 mRNA expression was markedly increased in CC tissues and cell lines (Figure 3(j,k)), and LOXL2 mRNA expression was not associated with the TNM stage of the tumors (Figure 3(l)). Notably, miR-195-5p expression was negatively correlated with circ_0000228 expression and LOXL2 mRNA expression in CC samples, and circ_0000228 expression and LOXL2 mRNA expression were positively correlated (Figure 3(m–o)). qRT-PCR and Western blot experiments confirmed that circ_0000228 knockdown remarkably augmented miR-195-5p expression and repressed LOXL2 mRNA expression in HCC94 and C33A cell lines; however, the transfection of miR-195-5p inhibitors partially counteracted the regulatory effects of circ_0000228 knockdown on miR-195-5p expression and LOXL2 expression (Figure 3(p,q)). The above findings validated that miR-195-5p was a downstream target of circ_0000228, and LOXL2 was a target gene of miR-195-5p, and circ_0000228 could positively regulated LOXL2 expression through repressing miR-195-5p in CC cells.

(a) A volcano plot was adopted to show the miRNAs that were remarkably up- and down-modulated in CC tissues compared with normal cervical tissue in the GEO dataset (GSE86100); (b) The Venn diagram was used to screen out the differentially expressed miRNAs in GSE86100, which contained complementary binding sites with circ_0000228; (c) Bioinformatics analysis indicated that high LOXL2 expression suggested poor clinical outcome of CC patients. (d) The complementary binding sites of circ_0000228 and miR-195-5p, miR-195-5p and LOXL2 3ʹUTR are presented, and the corresponding luciferase reporter vectors were designed; (E-F) Dual-luciferase reporter gene experiments confirmed that the binding sites between circ_0000228 and miR-195-5p, miR-195-5p and LOXL2 3ʹUTR were functional. (g) MiR-195-5p expression in 40 pairs of CC tissues/adjacent cervical tissues was detected by qRT-PCR; (h) MiR-195-5p expression in stage I and stage II CC patients was detected by qRT-PCR, respectively; (I) MiR-195-5p in HUCEC and CC cell lines (HeLa, HCC94, SW756, C33A) was detected by qRT-PCR.(j) LOXL2 mRNA expression in 40 pairs of CC tissues/adjacent cervical tissues was detected by qRT-PCR; (k) LOXL2 mRNA expression in HUCEC and CC cell lines (HeLa, HCC94, SW756, C33A) was detected by qRT-PCR; (L) LOXL2 mRNA expression in stage 1 and stage 2 CC patients was detected by qRT-PCR; (m-o) Pearson’s correlation analysis of the correlation relationships among circ_0000228 miR-195-5p, and LOXL2 expression in CC tissues; (p) qRT-PCR was employed to detect miR-195-5p expression after co-transfection with circ_0000228 siRNA and miR-195-5pin in CC cell lines; (q) qRT-PCR and Western blot experiments were employed to detect LOXL2 expression after co-transfection with circ_0000228 siRNA and miR-195-5p inhibitors in CC cell lines; All of the experiments were performed in triplicate. *P < 0.05, **P < 0.01 and ***P < 0.001.

### LOXL2 overexpression partially counteracted the tumor-suppressive effects of circ_0000228 knockdown on CC cells

**Figure 4.**
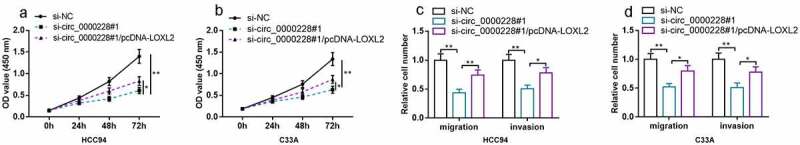
LOXL2 overexpression partially counteracted the inhibitory effect of knockdown circ_0000228 on CC cell multiplication, migration and invasion

To further validate that circ_0000228 exerted its biological functions in CC via regulating LOXL2, functional compensation experiments were executed. HCC94 cells and C33A cells were transfected with control siRNA, si-circ_0000228#1, or si_-circ_0000228#1 + LOXL2 overexpression plasmids. CCK-8 and Transwell experiments revealed that knocking down circ_0000228 remarkably impeded CC cell multiplication, migration, and invasion, while LOXL2 overexpression partially counteracted these effects (Figure 4(a–d)). The above experiments validated that circ_0000228 exerted an oncogenic effect in CC via regulating LOXL2.

(a,b) CCK-8 experiment was utilized to detect the multiplication of CC cells after the CC cells were co-transfected with circ_0000228 siRNA and LOXL2 overexpression plasmid. (c,d) Transwell experiment was executed to detect cell migration and invasion after the CC cells were co-transfected with circ_0000228 siRNA and LOXL2 overexpression plasmid.*P < 0.05 and **P < 0.01.

## Discussion

Accumulating research has reported that circRNAs are crucial regulators in CC development [18]. For instance, circ_0000069 enhances the growth, migration, and invasion of CC cells via miR-873-5p/TUSC3 axis [19]. circ_MYLK facilitates CC progression by up-modulating RHEB and activating the mTOR signaling pathway [20]. Circ_AKT1 accelerates CC progression by up-modulating AKT1 through miR-942-5p [21]. Circ-ZNF609 can promote the expression of E2F transcription factor 6 through competitively binding with miR-197-3p to facilitate the proliferation, migration and invasion of CC cells [22]. In this work, we identified that, circ_0000228, derived from ZEB1 transcript, which is transcribed from chromosome chr10:31661946–31676195, was up-regulated in CC tissues and cell lines. Functionally, we demonstrated that depletion of circ_0000228 repressed the growth and metastatic potential of CC cells. Our data suggest that circ_0000228 works as a novel oncogenic factor in CC, and it is a potential therapy target for CC treatment.

The ceRNA hypothesis provides a novel mechanism by which non-coding RNA participates in regulating the biological processes. CircRNAs can work as molecular sponge to indirectly modulate gene expression by decoying miRNAs [23]. In this work, circ_0000228 was revealed to be predominantly distributed in the cytoplasm of CC cells, so it is supposed that circ_0000228 may function as a ceRNA. Bioinformatics and dual-luciferase reporter gene experiments predicted and verified that miR-195-5p specifically and directly binds to circ_0000228. MiR-195-5p is reported to work as a tumor-suppressive factor that represses the progression of various tumors. Specifically, in prostate cancer, miR-195-5p represses cancer cell proliferation and angiogenesis by repressing the expression of proline-rich protein 11 [24]. In hepatocellular carcinoma, miR-195-5p targets MACC1 to block cell multiplication, migration, and invasion [25]. In ovarian cancer, miR-195-5p targets four and a half LIM domains 2 to restrain cell multiplication, migration and invasion [26]. In CC, miR-195-5p is reported to be remarkably down-modulated and it represses cancer cells’ growth and epithelial–mesenchymal transition (EMT) by targeting Yes1 associated transcriptional regulator [9]. The present study also demonstrated that miR-195-5p was remarkably down-modulated in CC tissues and cell lines, which is consistent with the previous report [9]. Also, the binding site between circ_0000228 and miR-195-5p was identified, and we demonstrated that circ_0000228 expression was negatively correlated with miR-195-5p expression in CC samples. Also, knocking down circ_0000228 in CC cell lines can markedly augmented miR-195-5p expression. These data indicate that circ_0000228 functions as a ceRNA to negatively regulate miR-195-5p in CC cells.

The primary function of LOX family members is to covalently cross-link elastin and collagen in the ECM to maintain the structural integrity of the tissue [27]. As a member of the LOX family, LOXL2 dysregulation is linked to the pathogenesis of several diseases, such as liver fibrosis and heart failure [28,29]. Reportedly, LOXL2 is also vital regulator in cancer biology. For instance, LOXL2 is remarkably up-modulated in colonic and esophageal cancers and its high expression is associated with unfavorable prognosis [30]. In gastric cancer, LOXL2 facilitates cancer cell metastasis through activating Src/FAK pathway [31]. In breast cancer, LOXL2 contributes to cancer progression through up-regulating the expression of vascular endothelial growth factor [32]. LOXL2 is also reported to be highly expressed in CC tissues, and is aberrant high expression is associated with the adverse prognosis of the patients, and functionally, LOXL2 facilitates the EMT of CC cells [11]. The current work validated that LOXL2 was remarkably up-modulated in CC tissues and cell lines. Additionally, LOXL2 was identified as a target gene of miR-195-5p, and it could be positively regulated by circ_0000228. Also, LOXL2 overexpression partially counteracted the suppressive effect of knocking down circ_0000228 on the proliferation, migration, and invasion of CC cells. These data partly explain the molecular mechanism of circ_0000228 dysregulation in CC, and provide evidence to support that circ_0000228 exerts an oncogenic effect in CC by modulating miR-195-5p/LOXL2 axis.

## Conclusion

Collectively, this work reports the expression characteristics and biological function of circ_0000228 in CC. Our findings suggest that circ_0000228/miR-195-5p/LOXL2 axis is a novel mechanism, which is involved in CC progression. Circ_0000228 may be a novel target for CC therapy. In the following work, the oncogenic function of circ_0000228 in CC should be further validated with animal models. Additionally, the other downstream targets and pathways of circ_0000228/miR-195-5p/LOXL2 axis remain to be explored.

## Highlights

- Circ_0000228 showed a remarkable up-modulation in CC tissues and cell lines
- Circ_0000228 knockdown impeded the growth and metastatic potential of CC cells
- Circ_0000228 facilitated CC progression through sponging miR-195-5p and increasing LOXL2 expression
